# Design and Testing of a Compliant ZTTΘ Positional Adjustment System with Hybrid Amplification

**DOI:** 10.3390/mi15050608

**Published:** 2024-04-30

**Authors:** Zhishen Liao, Zhihang Lin, Hui Tang, Bo Liu, Yingjie Jia

**Affiliations:** 1 State Key Laboratory of Precision Electronic Manufacturing Technology and Equipment, Guangdong University of Technology, Guangzhou 510006, China; 2112101123@mail2.gdut.edu.cn (Z.L.); 2112201428@mail2.gdut.edu.cn (B.L.); 1112201023@mail2.gdut.edu.cn (Y.J.); 2 School of Engineering, University of Warwick, Coventry CV4 7AL, UK

**Keywords:** spatial posture, flexure hinge, micro/nano adjustment, precision operation

## Abstract

This article presents the design, analysis, and prototype testing of a four-degrees-of-freedom (4-DoFs) spatial pose adjustment system (SPAS) that achieves high-precision positioning with 4-DoFs (Z/Tip/Tilt/Θ). The system employs a piezoelectric-driven amplification mechanism that combines a bridge lever hybrid amplification mechanism, a double four-bar guide mechanism, and a multi-level lever symmetric rotation mechanism. By integrating these mechanisms, the system achieves low coupling, high stiffness, and wide stroke range. Analytical modeling and finite element analysis are employed to optimize geometric parameters. A prototype is fabricated, and its performance is verified through testing. The results indicate that the Z-direction feed microstroke is 327.37 μm, the yaw motion angle around the X and Y axes is 3.462 mrad, and the rotation motion angle around the Z axis is 12.684 mrad. The x-axis and y-axis motion magnification ratio can reach 7.43. Closed-loop decoupling control experiments for multiple-input-multiple-outputs (MIMO) systems using inverse kinematics and proportional-integral-derivative feedback controllers were conducted. The results show that the Z-direction positioning accuracy is ±100 nm, the X and Y axis yaw motion accuracy is ±2 μrad, and the Z-axis rotation accuracy is ±25 μrad. Due to the ZTTΘ mechanism, the design proved to be feasible and advantageous, demonstrating its potential for precision machining and micro-nano manipulation.

## 1. Introduction

With the rapid development of the modern manufacturing industry, the accuracy requirements for ultra-precision machining and inspection are getting higher and higher. Submicron and even nanometer-level accuracy alignment systems are essential for various applications, such as wafer-level manufacturing and inspection, mass transfer and packaging of microminiaturized semiconductor products, robotic micro/nanomanipulations, and precision optical device polishing, etc. [1,2,3,4]. However, traditional high-precision motion systems with 3-DoFs (XYZ) fail to account for pitch, yaw, and small angular deviations [5]. These errors, at the spatial scale, pose critical challenges for the increasing miniaturization and integration of devices [6,7,8]. Moreover, relying solely on traditional flexure-based nanopositioning stages can provide submicron or nanometer accuracy, but it is hard to meet the large travel range requirements [9]. Therefore, how to develop a large-stroke nanopositioning system with multi-DoF levelling and deviation and compensation control function is critical to achieve the above advanced submicron or even nanoscale manufacturing.

To satisfy the need for ultra-high precision spatial pose adjustments, a few of motion systems have been proposed. According to the number of drive modules, these systems can be divided into two categories:

(1) Three-Branch Chain Parallel Systems: Three points determine a plane, and the plane’s position can be precisely controlled through the parallel connection of three chains. Although this system has a simple structure, the accuracy of a single-branched chain directly impacts the overall performance, necessitating the sacrifice of motion stroke (only a few tens of micrometers) to achieve high-precision single-branched chains [10,11,12,13,14].

(2) Multi-Branch Chain Differential Systems: Two or more non-intersecting lines form a surface. By manipulating two points that move independently along a straight line, the position of the line and the surface can be adjusted at intervals. This approach enhances accuracy by allowing for controllable differential and enables greater movement of the branch (up to centimeter level). However, the presence of redundant points makes the motion logic and mechanical structure more complicated, which can result in poor stability of the motion stage [15,16,17,18,19].

A lot of research on multi-degree-of-freedom motion stages has been carried out by previous researchers. In the design of stages, Zhang proposed an ingenious sinusoidal corrugated flexure linkage design, featuring structural symmetry and independent planar motion guidance for the two axes. With a stroke of approximately 130 μm per axis and maximum cross-talk below 2.5%, and a natural frequency of 590 Hz [20]. Zhang designed a nanopositioning stage employing the self-damping moving magnet actuator (SMMA) for long-stroke operation, supported by flexure guides. This system delivers 20 nm resolution within a ±5 mm motion range and maintains tracking errors below 0.1% of trajectory amplitudes at 1 Hz sinusoidal and triangular commands [21]. Yang designed a long-stroke nanopositioning stage with annular flexure guides and the classical feed-forward PID (FFPID) controller, achieving a ±5 mm motion range, 20 nm resolution, and 20 nm positioning accuracy at the maximum output position [22].

To improve the performances of stages further, many novel mechanisms and methods have been proposed. Li proposed Compliant Building Elements (CBE) to create practical flexure layouts, allowing early-stage design flexibility by assembling CBE blocks like constructing with LEGO bricks [23]. Niu introduced a corrugated dual-axial mechanism with structural symmetry, independent planar guidance for the two axes, stroke around 130 μm per axis, maximum cross-talk less than 2.5%, and an operating frequency of approximately 590 Hz [24]. Panas combined cross-pivot flexures to boost stiffness, load capacity, and range capacity in nanopositioning systems [25]. Al-jodah created a compact range three-degrees-of-freedom (3-DOF) micro/nanopositioning mechanism with leaf springs and voice coil motors (VCMs) [26]. Ling proposed an extended dynamic stiffness modeling approach for concurrent kinetostatic and dynamic analyses of planar flexure-hinge mechanisms with lumped compliance [27]. Moreover, the novel control and sensing strategies were proposed. Omidbeike presented a new sensing method that separately measures linear and angular displacements in multi-axis monolithic nanopositioning stages, providing enhanced accuracy [28]. Kuresangsai applied a linear time-varying (LTV) finite impulse response (FIR) prefilter to a flexure-based X-Y micro-positioning platform, significantly reducing settling times from over 6 s to just 0.4 s [29].

However, there are few stages that are suitable for direct application in the fields of wafer surface defect detection, Mini/MicroLED chip transfer packaging, etc., which need to balance large travel and high precision. Specifically, the three-branch chain stages have the advantage of ultra-high precision and are often used for nanoscale inspection but also have very small strokes, while the multibranch chain stages are suitable for processing large instruments. Therefore, there is a lack of a spatial pose adjustment system (SPAS) with hundred-micron motion stroke and hundred-nanometre accuracy to meet the needs of large-stroke, high-precision device fabrication and testing. Given accuracy as a priority, a three-branch parallel structure is advantageous for this type of stage. Therefore, the key to developing this posture adjustment system is to design a large-stroke and high-precision motion branch chain.

To cater for this requirement, this paper designed a ZTTΘ(Z/Tip/Tilt/Θ) SPAS with 4 DoFs. To 8-inch wafers or MiniLED backlight board as the object of application, the diameter of the device size is designed for 200 mm. due to the smaller the table height-to-diameter ratio, the higher the advantages of equipment integration and stability, this paper designs the device height of only 75 mm. Considering the need for high precision and large stroke the SPAS is designed based on a compliant structure with a simple and compact configuration, no motion friction [30,31]. The stage employs piezoelectric ceramics as actuators. To overcome the small stroke issue of piezoelectric ceramics, this paper proposes a bridge-lever composite compliant amplification mechanism. The high-precision motion of the designed SPAS is achieved by designing a MIMO PID controller. After performance testing, the Z-direction feed microstroke is 327.37 μm, the yaw motion angle around the X and Y axes is 3.462 mrad, and the rotation motion angle around the Z axis is 12.684 mrad. The Z-direction positioning accuracy is ±100 nm, the X and Y axis yaw motion accuracy is ±2 μrad, and the Z-axis rotation accuracy is ±25 μrad. Such stroke size and accuracy performance proves that the design is feasible and advantageous, demonstrating the potential to provide large-stroke, high-precision displacements and operations in precision operation areas such as wafer inspection and MiniLED chip transfer packaging.

The primary contribution of this work is the development of a compliant 4-DoFs system with hybrid amplification modules, which can achieve nanoscale positioning in 4-DoFs(Z/Tip/Tilt/Θ) to facilitate spatially accurate alignment. The rest of this paper is organized as follows: Section 2 presents the scheme design and operating principle of SPAS, which has been verified. Section 3 models the integrated ZTTΘ SPAS. Section 4 involves size optimization and finite element analysis. Validation experiments and performance evaluation are provided in Section 5. Finally, the achievements and further work are concluded in Section 6.

## 2. Configurations and Working Principle

### 2.1. Structure of the ZTTΘ SPAS

**Figure 1:**
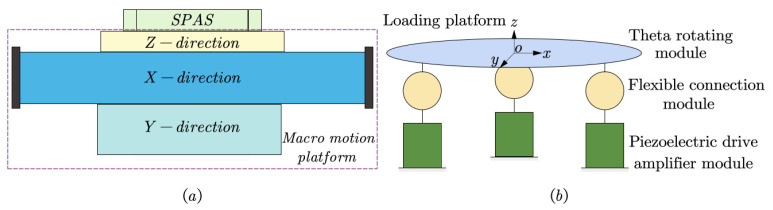
Design scheme of the ZTTΘ SPAS. (**a**) SPAS composite macro stage alignment system. (**b**) Working principle diagram.

As shown in Figure 1a, SPAS is designed as a precision-guaranteing device, compounded on a large-stroke 3-DoFs(XYZ) macro motion stage.As shown in Figure 1b the ZTTΘ SPAS consists of a Θ rotation module, three identical flexible connection modules, and three identical piezoelectric drive amplification modules. The stage’s operation is controlled by a combination of various motion branches, enabling it to achieve micro feed motion in the vertical direction, two spatial sway motions, and rotational micromotion around the vertical axis.

**Figure 2:**
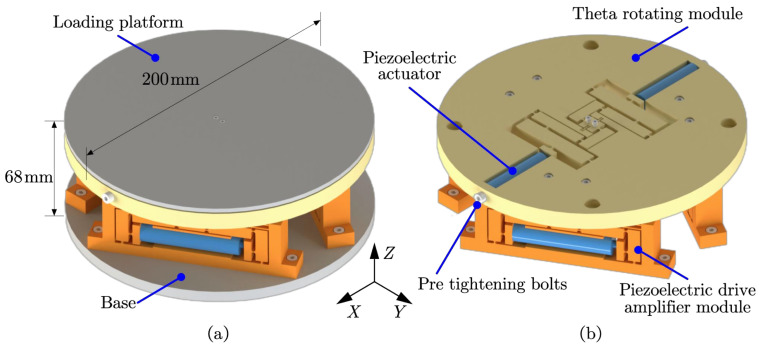
Structural design of the ZTTΘ SPAS based on flexure. (**a**) Overall schematic diagram. (**b**) Assembly diagram of each module.

The design configuration of the critical components of the SPAS is shown in Figure 2a,b. The stage mainly consists of the Θ rotation module and three parallel Z-axis motion branches. These branches are fixed in a 120° apart circular shape around the central axis of the positioning stage, with the base fastened to the Θ rotation module. The Θ rotation module is connected to the flexible connection module, which in turn is connected to the piezoelectric drive amplification module. This module is fixed to the base via bolts. The Θ rotation module is driven by two piezoelectric ceramic actuators, while each piezoelectric drive amplifier module is driven by a single piezoelectric ceramic actuator.

The ZTTΘ stage, presented in this paper, comprises 4-DoFs: a Z-axis feed micro motion, two spatial yaw motions (roll and pitch), and one spatial motion rotating in the vertical plane, with an overall diameter of 200 mm and a height of 68 mm.

### 2.2. Design of ZTT Module

**Figure 3:**
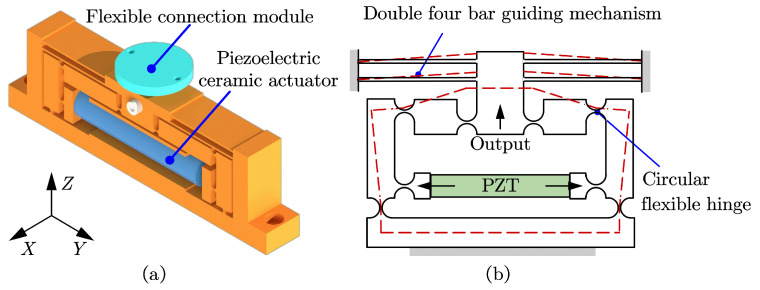
Design of piezoelectric drive amplifier module. (**a**) Piezoelectric drive amplifier module. (**b**) Principle of piezoelectric drive amplifier module.

This study employs a hybrid ZTT module integrating dual four-bar displacement guidance mechanisms and lever-bridge hybrid displacement amplification mechanisms. The piezoelectric amplification module of the SPAS is shown in Figure 3a. Although the design of the lever-type amplification mechanism is simple, its non-compact nature makes it prone to lateral parasitic displacement generation. Conversely, the bridge-type amplification mechanism exhibits a simple and compact structure, high displacement amplification factor, and good linear output displacement. However, it has low output stiffness and inadequate dynamic performance. The lever-bridge hybrid amplification mechanism, which combines the principles of lever amplification and bridge pressure rod instability, combines the benefits of a simple and compact structure, high displacement amplification factor, and good linear output displacement, while also improving the stiffness of the output end, thereby ensuring superior dynamic performance.

To improve the decoupling ability and output stiffness of the mechanism, a double four-bar displacement guidance mechanism is integrated at the end of the lever-bridge hybrid displacement amplification module. This parallel four-bar displacement guidance mechanism, utilizing the elastic deformation of the straight beam flexure hinge, enables approximate linear motion in the vertical direction at the output end. However, due to unavoidable coupling in the horizontal direction, this significantly compromises the positioning accuracy of the mechanism. The double four-bar displacement guidance mechanism exhibits a symmetrical structure with fixed ends, effectively eliminating coupling errors and ensuring minimal horizontal displacement loss. As shown in Figure 3b, the red dashed lines represent the motion and deformation of the designed module.

### 2.3. Design of Θ Module

**Figure 4:**
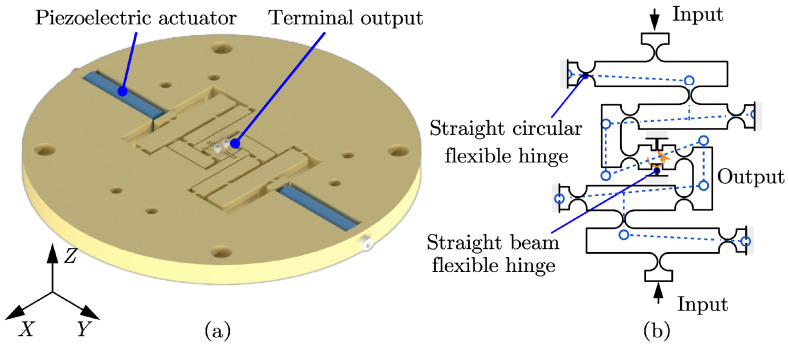
Design of Θ rotation module. (**a**) Oblique second survey view of Θ rotation module. (**b**) Principle of piezoelectric drive amplifier module.

The Θ rotation module of the SPAS, as shown in Figure 4a, utilizes a two-stage lever displacement amplification mechanism in combination with a straight beam flexure hinge. By initially inputting the displacement or force in the horizontal direction, the two-stage lever amplifies and generates rotational motion around the center point at the output end. Compared to a single output implementation of the rotating mechanism, the utilization of two symmetrical parallel-connected two-stage lever displacement amplification mechanisms enhances the stability of the Θ rotation module, resulting in improved stiffness for the entire module. This approach improves both the horizontal stability and stiffness of the stage.

The simultaneous application of an initial force or displacement to the output stage causes rotational motion around its center point. However, the design of the connecting rod leads to underconstrained DoFs in the non-rotational direction of the output stage, causing coupling errors. These errors include coupling along the X and Y axes in the horizontal plane, along the Z axis in the vertical plane, and rotational coupling around the X and Y axes in space. As shown in Figure 4b, the blue dashed lines represent the motion and deformation of the designed module.

To mitigate the coupling error at the output end of the Θ rotation module and enhance the motion precision of the mechanism, a symmetric straight beam flexure hinge is incorporated at the terminal output stage. This approach not only eliminates the coupling error but also enhances the output stiffness and motion accuracy, thereby ensuring the overall dynamic performance of the mechanism. Consequently, the working bandwidth of the mechanism is increased.

## 3. Modeling of ZTTΘ Stage

### 3.1. Static Modeling of ZTTΘ Stage

**Figure 5:**
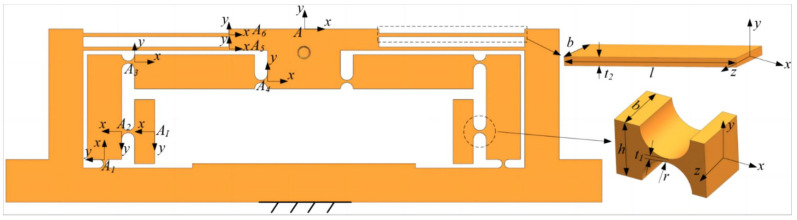
The local coordinate system of piezoelectric drive amplification module.

The symmetrical design of the piezoelectric-driven amplification module allows for the analysis of its left half initially. The comprehensive coordinate system and precise parameters are shown in Figure 5.

In the amplification module, the flexibility matrix $C_{A_{i}}$ (where i=1–6) represents the local flexibility matrix of point *i* in each $A_{i}$ element. When calculating the output compliance matrix, it can be deduced that Hinge 2 has no significant impact and can be disregarded at this stage. Therefore, the Flexure Hinges 1, 2, and 3 in the amplification module’s branch chain $A_{1}A_{3}A_{4}$ belong to series connections, and the local coordinate systems of Hinges 1, 3, and 4 are connected to $A_{i}-xy$ in the coordinate system $A_{4}-xy$. Converting the flexibility matrix of $A_{i}-xy$(i=1,3,4) to the coordinate system $A_{4}-xy$, it can be concluded that

(1) $$C_{A_{i}}^{A_{4}}=S_{i}^{4}R_{i}^{4}C_{A_{i}}{\left(R_{i}^{4}\right)}^{T}{\left(S_{i}^{4}\right)}^{T},\left(i=1,3\right)$$

where, $S(l_{i}^{j})$ denotes the translation matrix, $R_{i}^{j}$ denotes the rotation matrix [32]. when $l_{A_{i}A_{j},x}$ and $l_{A_{i}A_{j},y}$ (i=1–6, j=1–6) denote the length of the X and Y components of the displacement vector $A_{i}A_{j}$, respectively. They are expressed as

(2) $$S_{i}^{j}=S\left(l_{A_{i}A_{j}}\right)=\left[\begin{array}{ccc} 1 & 0 & l_{A_{i}A_{j},y}\\ 0 & 1 & -l_{A_{i}A_{j},x}\\ 0 & 0 & 1 \end{array}\right]$$

(3) $$R_{i}^{j}=R\left(\theta_{A_{i}A_{j}}\right)=\left[\begin{array}{ccc} \cos\left(\theta_{A_{i}A_{j}}\right) & -\sin\left(\theta_{A_{i}A_{j}}\right) & 0\\ \sin\left(\theta_{A_{i}A_{j}}\right) & \cos\left(\theta_{A_{i}A_{j}}\right) & 0\\ 0 & 0 & 1 \end{array}\right]$$

The series flexibility of Flexure Hinges 1, 3 and 4 in the coordinate system $A_{4}-xy$ of the branch chain $A_{1}A_{3}A_{4}$ is

(4) $$C_{A_{1}A_{3}A_{4}}^{A_{4}}=C_{A_{1}}^{A_{4}}+C_{A_{2}}^{A_{3}}+C_{A_{4}}$$

The flexibility matrix of the branch chain $A_{1}A_{3}A_{4}$ in the global coordinate system A−xy is:

(5) $$C_{A_{1}A_{3}A_{4}}^{A}=S_{A_{4}}^{A}C_{A_{1}A_{3}A_{4}}^{A_{4}}{\left(S_{A_{4}}^{A}\right)}^{T}$$

By transforming the flexibility matrix of the remaining free end local coordinate system $A_{i}-xy$ (i = 5,6) of the flexure hinge into the global coordinate system A−xy, it can be obtained that

(6) $$C_{A_{i}}^{A}=S_{i}^{A}C_{A_{i}}{\left(S_{i}^{A}\right)}^{T},\left(i=5,6\right)$$

The output flexibility matrix of the left half of the amplification module in the global coordinate system A−xy is influenced by the parallel connection between the dual four bar displacement guidance mechanism and the lever bridge hybrid displacement amplification mechanism in the amplification module:

(7) CoutAleft=CA1A3A4A−1+CA5A−1+CA6A−1−1

The piezoelectric-driven amplifier module exhibits a left-right symmetric structure. By counterclockwise rotation of the output flexibility matrix of the left half by 180 degrees, the output flexibility matrix of the right half can be obtained:

(8) CoutAright=R(π)leftCAiART(π)

Therefore, the output flexibility matrix for the piezoelectric drive amplification module, which utilizes a dual four-bar displacement guidance mechanism and a lever bridge hybrid displacement amplification mechanism within the global coordinate system A−xy, is presented as follows:

(9) CoutA=CAiAleft−1+CAiAright−1−1

The double four-bar displacement guidance mechanism on both sides of the output terminal stage of the amplification module is connected in parallel with the lever bridge hybrid displacement amplification mechanism. The stiffness model of the entire module is shown in Figure 5. The flexibility matrices of Flexure Hinge 1, 3, and 4 in the local coordinate system $A_{i}-xy$ (i=1,3,4) are transformed into the coordinate system $A_{2}-xy$ as shown below:

(10) $$C_{A_{i}}^{A_{2}}=R_{A_{i}}^{A_{2}}C_{A_{i}}{\left(R_{A_{i}}^{A_{2}}\right)}^{T}$$

The series flexibility matrix of the branch chain $A_{3}A_{4}$ and Flexure Hinges 3 and 4 in the coordinate system $A_{2}-xy$ is:

(11) $$C_{A_{3}A_{4}A_{5}A_{6}}^{A_{2}}=C_{A_{3}}^{A_{2}}+C_{A_{4}}^{A_{2}}+C_{A_{5}A_{6}}^{A_{2}}$$

The series flexibility matrix of the branch chain $A_{1}A_{3}A_{4}A_{5}A_{6}$ and Flexure Hinge 2 in the coordinate system $A_{2}-xy$ is

(12) $$C_{A_{i}}^{A_{2}}={\left({\left(C_{A_{3}A_{4}A_{5}A_{6}}^{A_{2}}\right)}^{-1}+{\left(C_{A_{1}}^{A_{2}}\right)}^{-1}\right)}^{-1}+C_{A_{2}}$$

The input flexibility matrix and the input stiffness of the piezoelectric drive amplification module are

(13) $$C_{in}^{A_{I}}=R_{A_{2}}^{A_{I}}C_{A_{i}}^{A_{2}}{\left(R_{A_{2}}^{A_{I}}\right)}^{T}$$

(14) $$K_{in}^{A_{I}}=\frac{1}{C_{in}^{A_{I}}}$$

**Figure 6:**
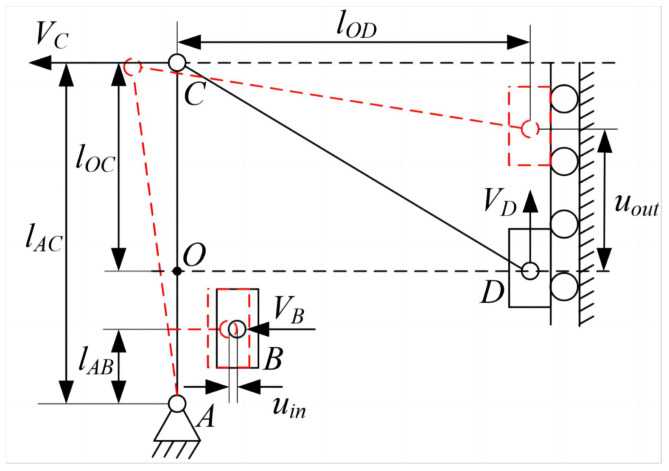
Simplified force analysis diagram.

As shown in Figure 6, the motion diagram of the left half of the piezoelectric drive amplification module is shown. The displacement amplification ratio, defined as the ratio of the output displacement to the input displacement, is derived from the compliance matrix and displacement relationship. Mathematically, the amplification ratio may be expressed as

(15) $$R_{A}=\frac{u_{out}}{u_{in}}=\frac{C_{out}}{C_{in}}\frac{l_{AB}l_{OC}}{l_{AC}\sqrt{{l_{OC}}^{2}+{l_{OB}}^{2}}}$$

**Figure 7:**
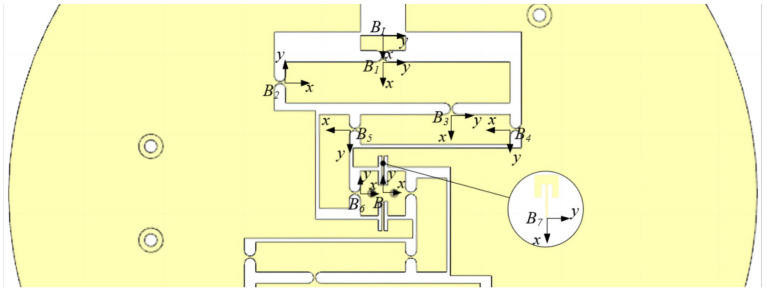
The local coordinate system of piezoelectric drive amplification module.

As shown in Figure 7, the established coordinate system reveals the parameter variables of its straight beam flexure hinge and straight circular flexure hinge, which exhibit similarities to those of the piezoelectric-driven amplification module. Among these, $C_{B_{i}}$ (i=1–7) denotes the local flexibility matrix for each point within the amplification module. Connected in parallel, the branch chain $B_{1}B_{2}B_{3}B_{4}B_{5}B_{6}B_{7}$ and straight beam flexure hinge form the upper half of the Θ rotation module in the global coordinate system B−xy. This yields the output flexibility matrix for the upper half of the module in the global coordinate system B−xy:

(16) CBiBup=CB1B2B3B4B5B6B−1+CB7B−1−1

Due to the symmetrical structure of the Θ rotation module, the output flexibility matrix of the upper half can be rotated 180 degrees counterclockwise to obtain the output flexibility matrix of the lower half:

(17) CBiBdown=R(π)upCBiBRT(π)

The output flexibility matrix of the Θ rotation module in the global coordinate system B−xy is

(18) CBiB=CBiBup−1+CBiBdown−1−1

The straight beam-type flexure hinges on both sides of the output terminal stage of the Θ rotation module are connected in parallel with the two-stage lever-type displacement amplification mechanism. The series flexibility matrix of the branch chain and Flexure Hinge 1 in the coordinate system $B_{I}-xy$ is

(19) $$C_{B_{i}}^{B_{1}}={\left({\left(C_{B_{3}B_{4}B_{5}B_{6}B_{7}}^{B_{1}}\right)}^{-1}+{\left(C_{B_{2}}^{B_{1}}\right)}^{-1}\right)}^{-1}+C_{B_{1}}$$

The input stiffness of the Θ rotation module is

(20) $$K_{B_{I}}=\frac{1}{C_{B_{i}}^{B_{I}}\left(1,1\right)}=\frac{1}{T_{B_{1}}^{B_{I}}C_{B_{i}}^{B_{1}}{\left(T_{B_{1}}^{B_{I}}\right)}^{T}}$$

**Figure 8:**
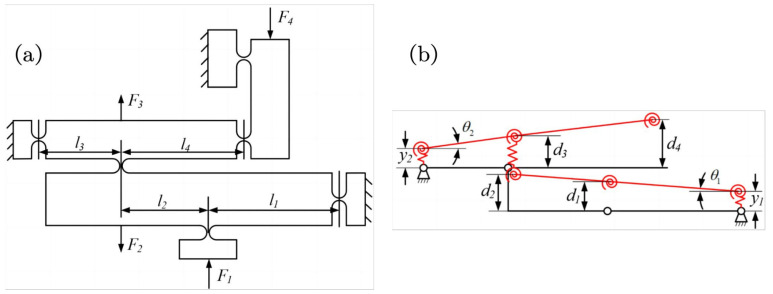
The Θ module. (**a**) Simplified force analysis diagram. (**b**) PRBM of Θ module.

As shown in Figure 8a, the dimensions of the two-stage lever displacement amplification mechanism within the Θ rotation module are presented. This mechanism exhibits a pronounced amplification effect, resulting in a substantial output displacement at the terminal stage. As shown in Figure 8b, the simplified analysis diagram of a two-stage lever-type displacement amplification mechanism reveals the straight circular flexure hinge at the fixed end as a rotating pivot. Its displacement is represented by a linear spring moving perpendicular to the rigid link and a rotating spring rotating around the pivot. During the actual calculation and analysis process, the deformation of the straight circular flexure hinge is calculated using the flexibility coefficient matrix. Subsequently, the displacement amplification ratio of the mechanism is obtained:

(21) $$R_{B}=\frac{d_{4}}{d_{1}}$$

### 3.2. Dynamic Modeling of ZTTΘ Stage

**Figure 9:**
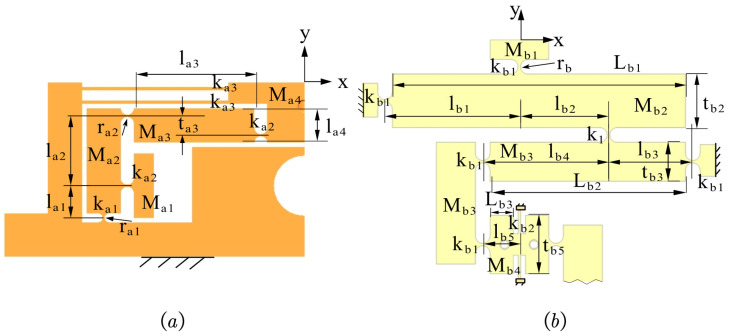
The local coordinate system of piezoelectric drive amplification module. (**a**) ZTT module. (**b**) Θ module.

This paper uses the Lagrange equation method to establish the kinematic differential equations of the ZTT module (marked A) and Θ module (marked B), respectively. Establish the dynamic model of the left half of the piezoelectric drive amplification module shown in Figure 9a, and the dynamic model of the upper part of the Theta rotation module shown in Figure 9b. Among them, by simplifying the straight circular flexure hinge and the straight beam flexure hinge into rotating torsion springs, the system is simplified as a spring-mass system. The kinetic energy of the system is generated by the movement of the rigid links in the mechanism, and the potential energy is generated by the elastic deformation of the straight circular flexure hinges and straight beam flexure hinges in the mechanism. The input displacement coordinate of the system is expressed as $\left(q_{1},q_{2}\right)$, $q_{1}$ is the displacement in the x-axis direction, $q_{2}$ is the displacement in the y-axis direction, and $m_{k}$ is the mass.

The potential energy of the system is the sum of the elastic potential energy generated by flexure hinges, which can be expressed as

(22) $$\begin{array}{cc} U_{A} & =\frac{1}{2}\sum_{k=1}^{6}k_{i,A}\varphi_{i,A}^{2}\\ \; & =\frac{k_{1}+k_{2}}{8l_{1}^{2}}q_{1}^{2}+\left[\frac{k_{2}R_{A}^{2}}{4\left[{\left(l_{3}+2r_{2}\right)}^{2}+t_{3}^{2}\right]}+\frac{k_{3}R_{A}^{2}}{l^{2}}\right]q_{2}^{2} \end{array}$$

(23) $$\begin{array}{cc} U_{B} & =\frac{1}{2}\sum_{k=1}^{7}k_{i,B}\varphi_{i,B}^{2}\\ \; & =\left[\frac{k_{1}}{l_{1}^{2}}+\frac{k_{1}{\left(l_{1}+l_{2}\right)}^{2}}{l_{1}^{2}l_{3}^{2}}+\frac{\left(2k_{1}+k_{2}\right){\left(l_{1}+l_{2}\right)}^{2}{\left(l_{3}+l_{4}\right)}^{2}}{2l_{1}^{2}l_{3}^{2}l_{5}^{2}}\right]q_{2}^{2} \end{array}$$

The kinetic energy of the system can be expressed as

(24) $$\begin{array}{cc} T_{A} & =\frac{1}{2}\sum_{k=1}^{4}m_{k,A}{\dot{q}}_{i,A}^{2}+\frac{1}{2}\sum_{k=2}^{3}J_{k,A}{{\dot{\varphi }}_{k,A}}^{2}\\ \; & =\left[\frac{1}{8}m_{1}+\frac{1}{32}m_{2}{\left(1+\frac{l_{2}}{l_{1}}\right)}^{2}+\right.\\ \; & \frac{1}{96}m_{2}\left[{\left(1+\frac{l_{2}}{l_{1}}\right)}^{2}+{\left(\frac{t_{4}}{l_{1}}\right)}^{2}\right]+\\ \; & \frac{1}{8}m_{2}{\left(\frac{1}{2}+\frac{l_{2}}{2l_{1}}+\frac{r_{1}}{l_{1}}\right)}^{2}\left.+\frac{1}{128}m_{3}{\left(1+\frac{l_{2}}{l_{1}}\right)}^{2}\right]{\dot{q}}_{1}^{2}+\\ \; & \left[\frac{1}{32}m_{3}R_{A}^{2}+\frac{1}{384}m_{3}\frac{R_{A}^{2}\left(l_{3}^{2}+l_{4}^{2}\right)}{{\left(l_{3}+2r_{2}\right)}^{2}+t_{3}^{2}}+\frac{1}{8}m_{4}R_{A}^{2}\right]{\dot{q}}_{2}^{2} \end{array}$$

(25) $$\begin{array}{cc} T_{B} & =\frac{1}{2}\sum_{k=1}^{4}m_{k,B}{\dot{q}}_{i,B}^{2}+\frac{1}{2}\sum_{k=2,3,5}^{3}J_{k,B}{{\dot{\varphi }}_{k,B}}^{2}\\ \; & =\left[\frac{1}{2}m_{1}+\frac{1}{24}m_{2}\frac{L_{2}^{2}+t_{2}^{2}+6{\left(L_{2}+2r\right)}^{2}}{l_{1}^{2}}+\right.\\ \; & \frac{1}{8}m_{3}\frac{{\left(l_{1}+l_{2}\right)}^{2}{\left(L_{3}+2r\right)}^{2}}{l_{1}^{2}l_{3}^{2}}+\\ \; & \frac{1}{24}m_{3}\frac{{\left(l_{1}+l_{2}\right)}^{2}\left[3{\left(L_{3}+2r\right)}^{2}+L_{3}^{2}+t_{3}^{2}\right]}{l_{1}^{2}t_{3}^{2}}+\\ \; & \left.\frac{1}{2}m_{4}\frac{{\left(l_{1}+l_{2}\right)}^{2}{\left(l_{3}+l_{4}\right)}^{2}}{l_{1}^{2}l_{3}^{2}}\right]{\dot{q}}_{2}^{2}+\\ \; & \frac{1}{24}m_{5}\frac{{\left(l_{1}+l_{2}\right)}^{2}{\left(l_{3}+l_{4}\right)}^{2}\left(t_{5}^{2}+4L_{5}^{2}\right)}{l_{1}^{2}l_{3}^{2}l_{5}^{2}} \end{array}$$

The kinetic energy and potential energy of the system can be substituted into the Lagrange equation:

(26) $$\frac{d}{dt}\left(\frac{\partial L}{\partial {\dot{q}}_{i}}\right)-\frac{\partial L}{\partial q_{i}}=0\left(i=1,2\right)$$

In the formula, L=T−U. Obtain the undamped free vibration frequency of the piezoelectric drive amplification module:

(27) $$f=\frac{w_{n}}{2\pi }=\frac{1}{2\pi }\sqrt{\frac{K}{M}}$$

## 4. Optimization and FEA

### 4.1. Multi-Objective Optimization

According to statics and dynamics models, the main dimensional parameters of the piezoelectric-driven amplification module are the width *b* of the straight beam, the dimensions of the straight-circular flexible hinge ($r_{c},t_{c}$), the dimensions of the straight-beam flexible hinge ($l_{s},t_{s}$), and the dimensions of the lever-bridge hybrid displacement amplification mechanism ($l_{AB},l_{AC},l_{OC},l_{OD}$), as shown in Figure 5. Therefore, to design the performance parameters of the SPAS, essentially determining each of the key parameters, this can be viewed as a multi-objective optimization problem where the optimization objective is the displacement of the output, and therefore the objective function can be set to the amplification ratio as shown in Equations (28) and (29).The main module dimensions of SPAS are optimized using a Genetic Algorithm.

(1) ZTT module: Limited by the dimensions of the piezoelectric ceramics, the overall thickness $b_{a}$ is set at 18 mm. Meanwhile, to ensure the Z-axis stiffness of the piezoelectric drive amplification module, $t_{a2}$ is set to 0.8 mm. The remaining seven dimensional parameters ($r_{a},t_{a1},l_{a},l_{AB},l_{AC},l_{OC},l_{OD}$) are used as design variables.

(28) $$\max u_{out}=\frac{K_{p1}}{K_{p1}+K_{A_{I}}}r_{p1,0}R_{A}$$

where $u_{out}$ is the output displacement, $K_{A_{I}}$ is the input stiffness of the amplifier module, and $R_{A}$ is the amplification ratio of the module, which are derived from the kinematic modeling in the Section 3. $K_{p1}$ and $r_{p1,0}$ are the stiffness and nominal stroke of the selected piezoelectric ceramic PSt150/10/60 VS15, respectively.

Taking into account the module’s overall size, height, and wire cutting gap, the range of design variables and the obtained optimal solution are given in Table 1. The displacement amplification ratio is 7.92, and the maximum displacement of the theoretical output is 323.29 μm.

(2) Θ module: As shown in Figure 7, taking into account the dimensions of the piezoelectric ceramics, as well as the compactness of the overall dimensions, the partial parameters are determined: $b_{b}=12$ mm, $l_{b}=7.8$ mm, $l_{b5}=7.5$ mm, $t_{b2}=11$ mm, $t_{b3}=8$ mm, and $t_{b5}=12$ mm. The remaining seven dimensional parameters ($r_{b}$, $t_{b1}$, $l_{b1}$, $l_{b2}$, $l_{b3}$, $l_{b4}$) are used as design variables. Similarly, to achieve a larger output rotation angle, the displacement amplification ratio of the Θ rotation module from kinematic modeling in Section 3 is selected as the optimization objective:

(29) $$\max R_{B}=\frac{d_{4}}{d_{1}}$$

The size parameters of the six design variables and the obtained optimal solution are given in Table 2. And the amplification ratio is 3.24.

### 4.2. Simulation

The model of SPAS is established by the 3D design software UG NX10.0. The static and dynamic characteristic are analyzed by the finite element analysis software ANSYS Workbench 19.0.

In FEA, the fineness of meshing affects the accuracy of the simulation, and the key to the performance of the flexible mechanism SPAS is the flexible hinge, in order to improve the accuracy of the simulation, the mesh size of the flexible hinge is set to 0.5 mm, and in order to improve the speed and stability, the mesh size of the rigid body is set to 1.5 mm. The material used in this study is AL7075-T6 (elasticity modulus E=71.7 GPa, Poisson’s ratio μ = 0.33, density ρ = 2810 kg/m^3^, Yield strength σ = 503 MPa). The FEA results of the piezoelectric driven amplifier module and the Θ rotation are shown in Table 3 and Table 4, respectively.

**Figure 10:**
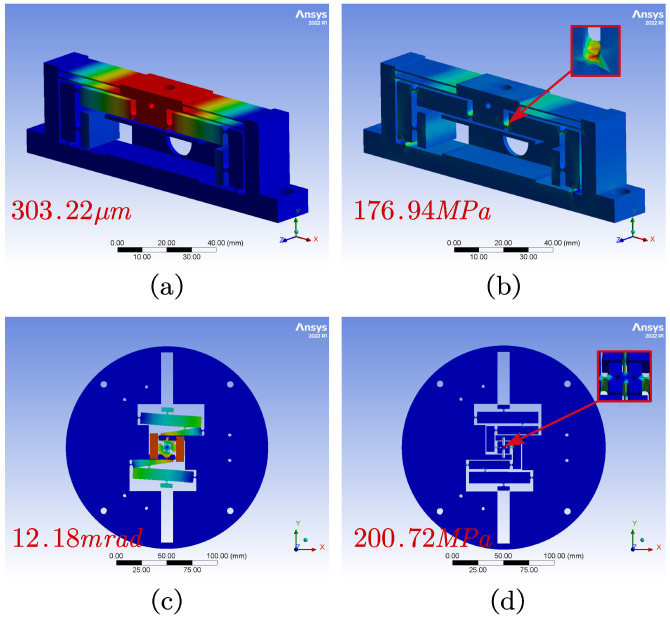
FEA results. (**a**) Maximum output displacement of the piezoelectric-driven amplifier module. (**b**) Maximum bending stress of the amplifier module. (**c**) Maximum output rotation angle of the Θ rotation module. (**d**) Maximum bending stress of Θ rotation module.

As shown in Figure 10a,b, the maximum output displacement of the piezoelectric driven amplifier module is 303.22 μm, the ratio is 7.43, and the maximum bending stress is 176.94 MPa. As shown in Figure 10c,d, the maximum output rotation angle of the Θ rotation module is 12.1789 mrad, the ratio is 2.98, and its maximum bending stress is 200.72 MPa, which is far less than the allowable limit stress of material AL7075-T6 of 503 MPa. These results indicate that the designed modules will not undergo fatigue failure within the normal working range, thereby fulfilling the design specifications.

**Figure 11:**
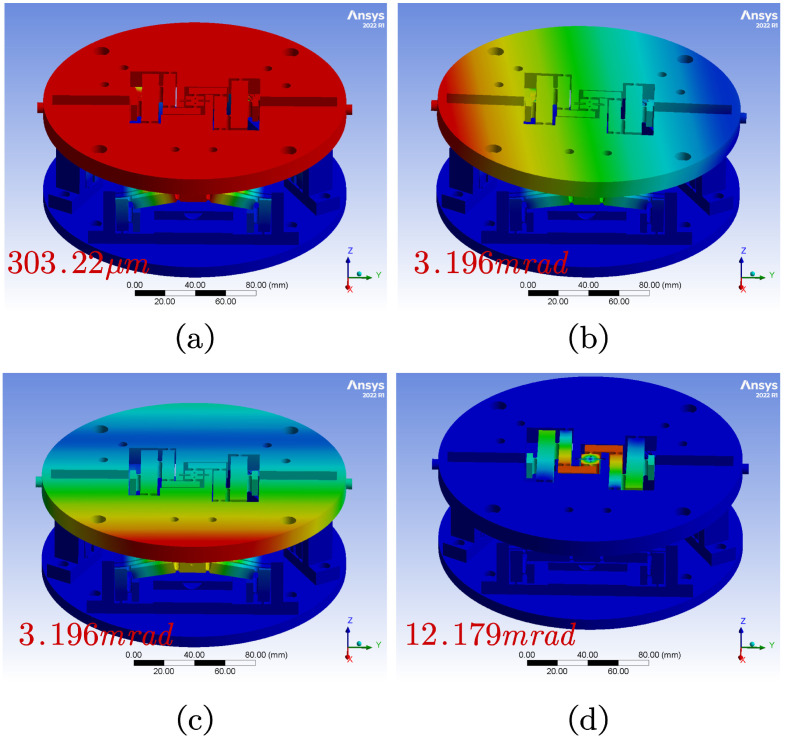
FEA results. (**a**) Maximum output displacement of Z-axis. (**b**) Maximum output yaw angle around the X axis. (**c**) Maximum output yaw angle around the Y axis. (**d**) Maximum output rotational angle around the Z axis.

As shown in Figure 11a, when the identical displacement excitation is applied to the three Z-axis motion branches, the terminal motion stage generates an overall Z-axis feed motion, with the simulated output Z-axis displacement reaching 303.22 μm. In Figure 11b,c, only one of the Z-direction motion branches is subjected to displacement excitation, while the remaining two Z-direction motion branches are in a free state. Consequently, the terminal motion stage produces yaw motion around the X and Y axes, with the simulation value of the output swing angle being 3.196 mrad. In Figure 11d, applying symmetrical displacement excitation to the Θ rotation module results in rotational motion around the Z-axis, with the simulated output rotation angle value reaching 12.179 mrad. The simulation coupled errors of the SPAS are shown in Table 5, the results indicated that because of the symmetrical mechanism design, the coupled errors are very small.

**Figure 12:**
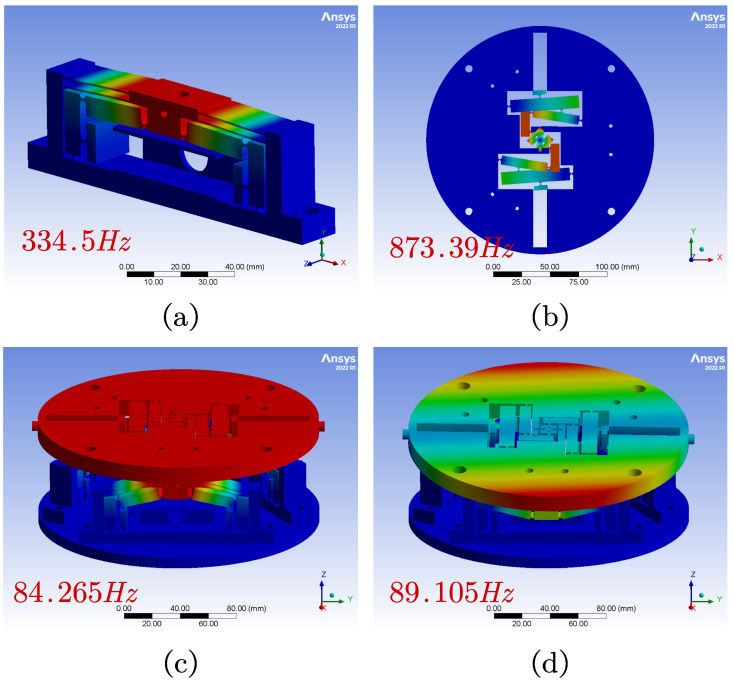
FEA results. (**a**) Natural frequency of the amplifier module. (**b**) Natural frequency of the Θ rotation module. (**c**) First frequency of the ZTTΘ stage. (**d**) Second frequency of the ZTTΘ stage.

As shown in Figure 12a, the initial natural frequency of the module is 334.5 Hz, indicating its motion state during routine operation. In Figure 12b, the Θ rotation module’s initial natural frequency is 873.39 Hz, signifying its motion state under routine operation. The first mode deformation in both figures exhibits yaw motion, with the first mode in Figure 12c revolving around the Y-axis and the second mode in Figure 12d revolving around the X-axis. Their respective natural frequencies are 84.265 Hz and 89.105 Hz. The results show that the designed device has good dynamic response performance.

## 5. Experiment and Discussion

### 5.1. Experimental Setup

**Figure 13:**
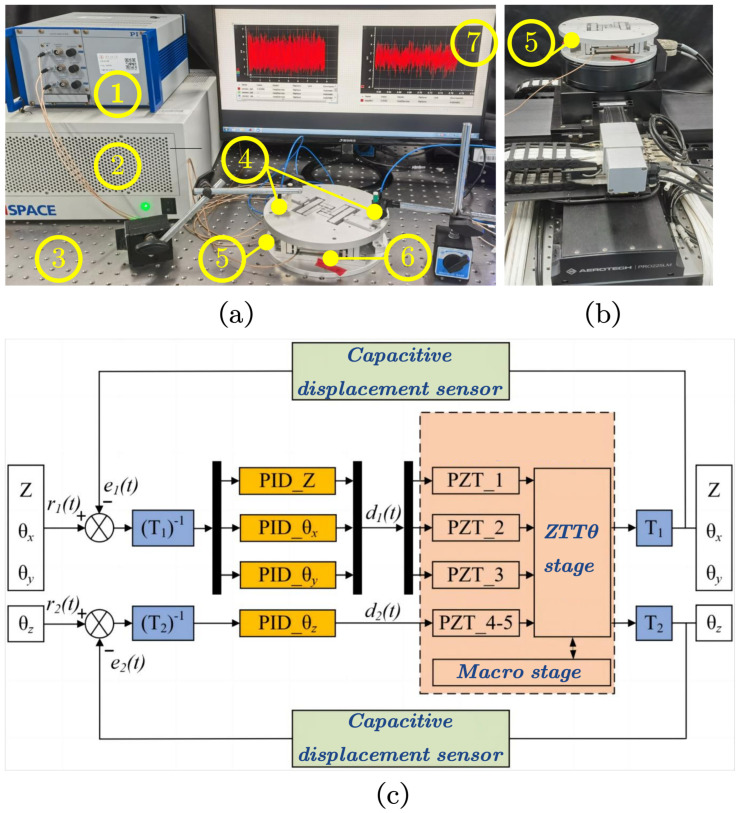
The 4-DoFs SPAS. (**a**) (1) PZT voltage amplifier, (2) controller system, (3) vibration isolation stage, (4) displacement sensor, (5) ZTTΘ stage, (6) macroactuating PZT, (7) PC host system. (**b**) Aerotech macromotion stage. (**c**) Schematic diagram of control method.

As shown in Figure 13a, a prototype 4-DoFs SPAS is fabricated. The system consists of an AL7075-T6 material–based SPAS and is driven by three piezoelectric ceramic actuators (XMT,PSt150/10/60 VS15) for ZTT module and two for Θ module. The measurement system comprises three capacitive displacement sensors (SYMC, NS-DCS14), deployed above the three Z-direction motion branches, to detect the SPAS’s motion position. The system is mounted on the Winner Optics (WN01AL) stage to mitigate external vibrations caused by environmental factors.

The decoupling of multiple–input–multiple–output (MIMO) control in the system is accomplished using inverse kinematics and a closed-loop PID control strategy. The control strategy of the 4-DoFs SPAS is shown in Figure 13c. The input of the entire control system comprises the reference Z-direction feed micromotion, yaw motion around the X-axis and Y-axis, and rotational motion around the Z-axis. After inverse kinematics analysis, the corresponding driving voltage signals $d_{1}\left(t\right)$ and $d_{2}\left(t\right)$ are output through the closed-loop controller, thereby driving the ZTTΘ. The SPAS utilizes a capacitive displacement sensor measurement system to detect the actual displacement signal of the stage in real-time, and compare it with the reference signals $r_{1}\left(t\right)$ and $r_{2}\left(t\right)$. Subtracting yields an error value $e_{1}\left(t\right)$ and $e_{2}\left(t\right)$. The four axes are adjusted by their respective PID controllers to achieve closed-loop motion control of the four axes.

### 5.2. Open-Loop Test

**Figure 14:**
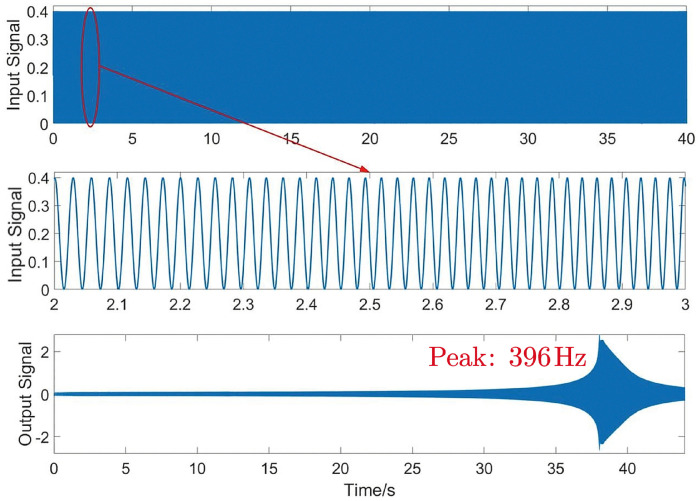
Sweep signal experiment of piezoelectric driven amplifier module.

**Figure 15:**
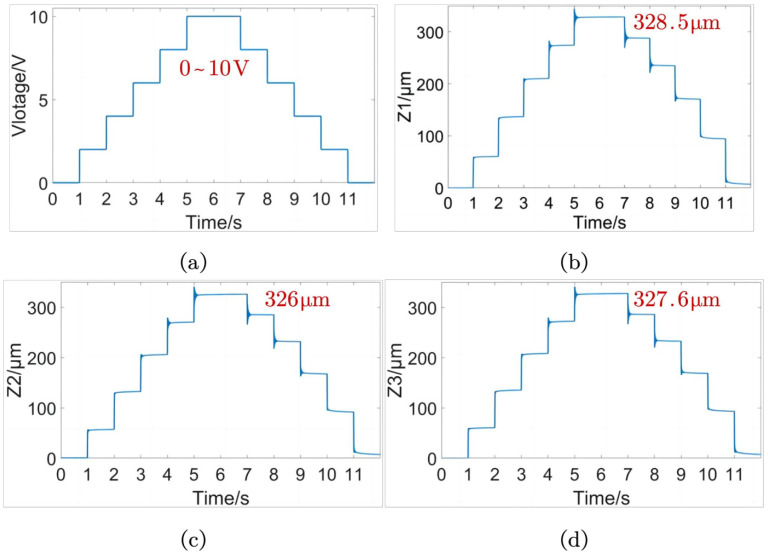
Open-loop step signal test diagram of piezoelectric drive amplifier module. (**a**) Input step voltage signal of piezoelectric actuator. (**b**) Output signal of Z1 module displacement. (**c**) Output signal of Z2 module displacement. (**d**) Output signal of Z3 module displacement.

As shown in Figure 14, the first-order natural frequency obtained by sinusoidal frequency sweeping of the amplification module is 396 Hz, which is similar to the result of 334.5 Hz obtained by simulation. The ZTTΘ workspace encompasses the Z-direction feed micro motion stroke, the yaw motion stroke, and the rotation motion stroke. As shown in Figure 15a, the step voltage signals (0–10 V) applied to the three piezoelectric drive amplification modules are amplified by a piezoelectric ceramic voltage driver by a factor of 15. The resulting output terminal displacements are measured by capacitive displacement sensors.

In Figure 15b–d, we can see that the output displacement stroke of Module 1 is 328.5 μm, Module 2 is 326 μm, and Module 3 is 327.6 μm. It is clear that the output displacement of the three modules is almost identical. By using the displacement transformation relationship, we can calculate that the actual Z-direction feed microstroke of the ZTTΘ SPAS is 327.37 μm. Additionally, the actual output yaw angle is 3.462 mrad.

### 5.3. Closed-Loop Test

**Figure 16:**
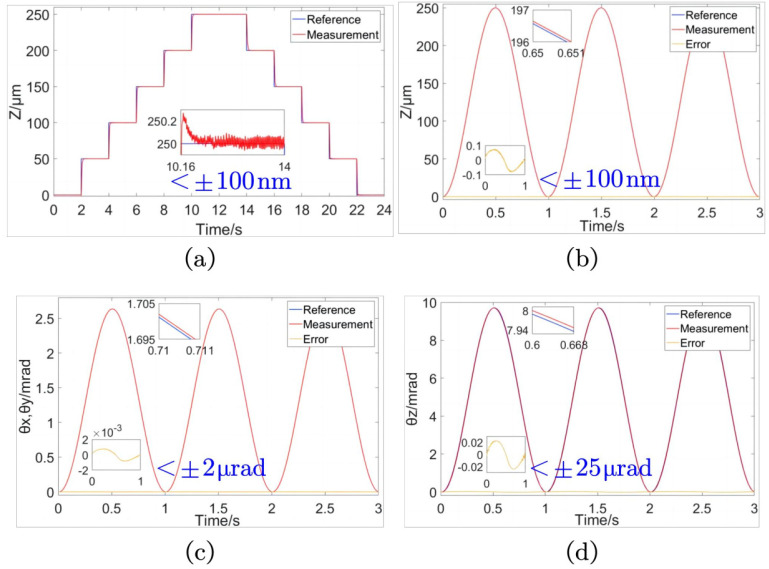
Closed-loop test diagram of the positioning stage. (**a**) Trace diagram of the ladder signal of the Z-direction motion. (**b**) Trace diagram of the sinusoidal signal of the Z-direction motion. (**c**) Trace diagram of the sinusoidal signal of the yaw motion. (**d**) Trace diagram of the sinusoidal signal of the Rotational motion.

The closed-loop motion tracking experiment of the SPAS is designed to assess its performance. The control model is established using MATLAB 2022b/Simulink and compiled into dSPACE for calculation. The basic sampling time is 0.1 μs, and the PID controller parameters are specified as shown in Table 6. To evaluate the stage’s performance, tracking control experiments are conducted on conventional Z-direction motion signals (Figure 16a). The reference signal is shown in Table 7, lasting 2 s. The maximum steady-state error after each step is stabilized within 100 nm, resulting in a closed-loop positioning accuracy of 100 nm for the step signal.

As shown in Figure 16b, a tracking test is performed on the Z-direction closed-loop sine signal of the positioning stage. The reference signal is shown in Table 7. The maximum steady-state error of the trajectory tracking is kept within ±100 nm, thus ensuring uniform ZTTΘ positioning accuracy of ±100 nm for the Z-direction feed micro motion of the SPAS. Similarly, as shown in Figure 16c, a tracking test is conducted on the closed-loop sine signal of the positioning stage’s yaw motion. The reference signal is shown in Table 7. The maximum steady-state error of the trajectory tracking is kept within ±2 μrad, thus allowing for the determination of the closed-loop positioning accuracy of the SPAS’s yaw motion around the X-axis and Y-axis to be ±2 μrad. Lastly, as shown in Figure 16d, a tracking test is performed on the closed-loop sine signal of the positioning stage’s rotational motion. The reference signal is shown in Table 7. The maximum steady-state error of the trajectory tracking is kept within ±25 μrad, ensuring uniform ZTTΘ positioning accuracy of ±25 μrad for the SPAS rotating around the Z-axis.

### 5.4. Performance Evaluation and Discussions

In high-precision operations and manufacturing, such as MicroLED chips, accurate alignment of spatial position during transfer, packaging, inspection, and repair processes is crucial for ensuring yield. To address this requirement, this paper presents a high-precision 4-DoFs (ZTTΘ) SPAS. An experimental evaluation is conducted on the system, demonstrating precise positioning of four degrees of freedom in space, as evidenced by the performance parameter test results reported in Table 8.

These results were obtained through a series of trajectory tracking test experiments, demonstrating that ZTTΘ SPAS can effectively adjust pose in large stroke, high-precision, and MDOF space, including yaws, lifting, rotation, and more. This capability makes ZTTΘ SPAS suitable for use in Micro LED display equipment manufacturing and testing. Furthermore, topology and module optimization theories [33] can be employed to optimize the device’s strength and quality, thereby enhancing its dynamic performance. This work contributes to the advancement of high-precision operation and manufacturing, which underscores the importance of precise alignment during various processes.

## 6. Conclusions

This paper presents the design, analysis, and prototype development of a 4-DoFs SPAS. The main achievements are summarized as follows:

(1) Designed, optimized, and manufactured a ZTT module that combines a bridge-type lever hybrid amplification with a dual four-bar guide mechanism, and a multi-stage lever rotation Θ module. Compared with the traditional piezoelectric drive mechanism, it exhibits low coupling, high stiffness, and large stroke.
(2) Designed and built a compliant 4-DoFs SPAS driven by piezoelectric ceramics, which can realize four high-precision movements of Z-axis lifting, Tip, Tilt, and rotation.
(3) With the developed system and controlling strategy, the experiments including open-loop test, closed-loop performance test, accuracy and travel range tests are successfully carried out in detail.

The experimental results indicate that the Z-direction stroke is 327.37 μm, the tip-tilt value is 3.462 mrad, and the yaw value around the Z axis is 12.684 mrad. The closed-loop positioning accuracy is ±100 nm, the tip−tilt is ±2 μrad, the yaw around the Z-axis is ±25 μrad. In conclusion, the relevant theoretical derivations and experimental results fully demonstrate that the developed system can well meet the advanced submicron and nanoscale manufacturing requirements.

## Figures and Tables

**Table 1:** The value of optimization variable of the piezoelectric driven amplifier module.

Size (mm)	$r_{a}$	$t_{a1}$	$l_{a}$	$l_{\mathrm{AB}}$	$l_{\mathrm{AC}}$	$l_{\mathrm{OC}}$	$l_{\mathrm{OD}}$
range	[0.5, 2]	[0.3, 0.8]	[32, 36]	[5, 9]	[20, 25]	[3, 6]	[26, 35]
best	1.5	0.5	34	7.5	23.75	4.5	31

**Table 2:** The value of optimization variable of the Θ rotation module.

Size (mm)	$r_{b}$	$t_{b1}$	$l_{b1}$	$l_{b2}$	$l_{b3}$	$l_{b4}$
range	[0.5, 2]	[0.3, 0.8]	[24, 29]	[16, 20]	[15, 19]	[23, 27]
best	1.5	0.5	27.5	18.25	17.25	25.75

**Table 3:** The FEA results of the piezoelectric driven amplifier module.

F/N	$u_{\mathrm{in}}/\mu$m	$u_{\mathrm{out}}/\mu$m	$R_{A}$	$\sigma_{\max}$/MPa	Error/%
100	10.28	76.35	7.43	44.47	6.19
200	20.45	151.98	7.43	88.94	6.19
300	30.63	227.59	7.43	133.41	6.19
400	40.81	303.22	7.43	176.94	6.19

**Table 4:** The FEA results of the Θ rotation.

F/N	$u_{\mathrm{in}}/\mu$m	$u_{\mathrm{out}}/\mu$m	$R_{A}$	$\sigma_{\max}$/MPa	Error/%
60	11.45	34.12	2.98	73.16	8.02
90	17.18	51.19	2.98	109.75	8.02
120	22.90	68.25	2.98	146.33	8.02
150	31.42	93.65	2.98	200.72	8.02

**Table 5:** Deformation and coupled error.

Directions	Deformation Wanted	Coupled Error	Percentage
*Z* aixs	303.22 μm	0.14 μm	0.004%
$\theta_{x}$ and $\theta_{y}$	3.196 mrad	0.001 mrad	0.003%
$\theta_{z}$	12.179 mrad	0.012 mrad	0.01%

**Table 6:** PID controller parameters.

Directions	$K_{p}$	$K_{i}$	$K_{d}$
*Z* aixs	3	350	0.0055
$\theta_{x}$ and $\theta_{y}$	2.95	299	0.0045
$\theta_{z}$	0.32	225	0.0003

**Table 7:** Reference sinusoidal signal.

Directions	Aptitude	Frequency
*Z* aixs	250 μm	1 Hz
$\theta_{x}$ and $\theta_{y}$	2.6 mrad	1 Hz
$\theta_{z}$	9.7 mrad	1 Hz

**Table 8:** Performance parameters test results of the ZTTΘ SPAS.

Items	Strokes	Accuracy
Direction of *Z* aixs	327.37 μm	±100 nm
Deflection of $\theta_{x}$ and $\theta_{y}$	3.462 mrad	±2 μrad
Rotation of $\theta_{z}$	12.684 mrad	±25 μrad

## Data Availability

Data available on request from the authors.
